# Scar endometriosis: Looking beyond the diagnosis - A case series

**DOI:** 10.4102/sajr.v26i1.2493

**Published:** 2022-11-29

**Authors:** Stuti Chandola, Anju Garg

**Affiliations:** 1 Department of Radiodiagnosis, Maulana Azad Medical College, New Delhi, India

**Keywords:** scar endometriosis, perineal scar endometriosis, abdominal wall endometriosis

## Abstract

Scar endometriosis usually affects the abdominal wall or the perineum. Virtually all cases are linked with some form of surgical manipulation. Although the clinical diagnosis of scar endometriosis may be straightforward with classical symptomology, imaging with ultrasound and MRI are important for the determination of its extent, which is imperative for adequate preoperative planning. In addition, assessment of perineal scar endometriosis also requires the identification of anal sphincter complex involvement, which can significantly impact the surgical approach. Radiology plays a vital role in its diagnosis in atypical clinical scenarios.

**Contribution:**

This series of four cases describes the morphology and highlights the importance of imaging in the surgical management of scar endometriosis; three with abdominal wall involvement and one with the involvement of perineum.

## Introduction

Endometriosis is a multifocal and polymorphic disease in the reproductive age group with a prevalence of 10% – 15%^1^; however, implantation of endometriotic tissue into scar sites remains an infrequent entity to date. Possible sites include the abdominal wall and perineum with involvement of the skin, subcutaneous tissue and underlying musculature. Surgery remains a sine qua non inciting factor for this condition and consequently, Cesarean section (CS) scars in the anterior abdominal wall constitute the most common site, with an estimated incidence of approximately 0.03% – 0.4%.^2,3^ Scar endometriosis has also been reported in post-hysterotomy scars, perineal episiotomies, post-Bartholin’s gland excisions, in laparoscopic trocar tracts and even in amniocentesis needle tracts.^3^ Involvement of the scar in the uterus is an even more rare entity, with limited case reports described in literature.^4^

With the aim of highlighting the importance of imaging in the assessment of scar endometriosis, four patients are presented, all of whom were provisionally diagnosed with scar endometriosis through a combination of clinical and radiological features with subsequent pathological confirmation. Besides suggesting the diagnosis, imaging plays an unequivocal role in evaluating the depth of infiltration by these lesions, which is imperative for preoperative planning and management.

## Case series

Four patients from the outpatient department were included in the series. Three patients had anterior abdominal wall involvement and one patient had involvement of perineum at the episiotomy site. Informed consent was obtained and relevant clinical details including surgical history were recorded in each case.

The radiological assessment in all patients involved a multimodality imaging approach with ultrasound (transabdominal or transperineal), including colour Doppler, followed by MRI on a 3T scanner. Ultrasound scanning especially involved the use of a high frequency transducer (7 MHz to 10 MHz) as the lesions were not readily apparent on examination with the routine 3 MHz – 5 MHz transducer.

When planning the MRI, it was ensured that the anterior saturation bands were cautiously placed to avoid concealing lesions. Conventional MR sequences were then acquired, which included T1W, T2W and fat saturated T1W sequences in the sagittal and axial planes. This was followed by acquisition of diffusion weighted images (*b*–values = 0 mm^2^/s, 400 mm^2^/s, 800 mm^2^/s; with corresponding ADC maps) and post-contrast imaging sequences in the axial and sagittal planes following administration of intravenous gadolinium at a dose of 0.1 mmol/kg.

### Case 1

A 36-year-old lady presented with complaints of progressively increasing periodic pain and swelling near her abdominal scar coinciding with her menstrual cycles for a period of 3-years. She had undergone sling surgery (cervicopexy) for uterine prolapse five years previously. Clinical examination demonstrated a well-approximated and healed Pfannenstiel incision scar with no evidence of erythema or drainage. A firm tender palpable mass, 2 cm × 1.5 cm was situated superior and lateral to the scar region on the left.

**FIGURE 1:**
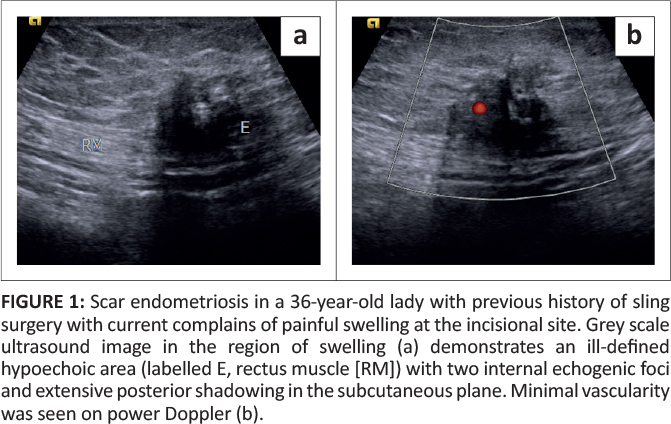
Scar endometriosis in a 36-year-old lady with previous history of sling surgery with current complains of painful swelling at the incisional site. Grey scale ultrasound image in the region of swelling (a) demonstrates an ill-defined hypoechoic area (labelled E, rectus muscle [RM]) with two internal echogenic foci and extensive posterior shadowing in the subcutaneous plane. Minimal vascularity was seen on power Doppler (b).

**FIGURE 2:**
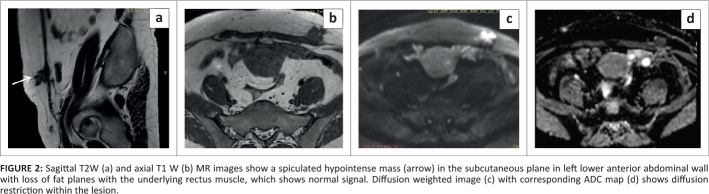
Sagittal T2W (a) and axial T1 W (b) MR images show a spiculated hypointense mass (arrow) in the subcutaneous plane in left lower anterior abdominal wall with loss of fat planes with the underlying rectus muscle, which shows normal signal. Diffusion weighted image (c) with corresponding ADC map (d) shows diffusion restriction within the lesion.

**FIGURE 3:**
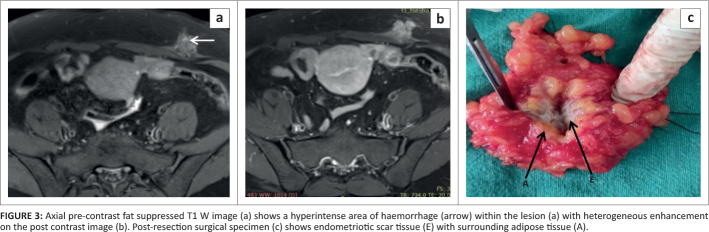
Axial pre-contrast fat suppressed T1 W image (a) shows a hyperintense area of haemorrhage (arrow) within the lesion (a) with heterogeneous enhancement on the post contrast image (b). Post-resection surgical specimen (c) shows endometriotic scar tissue (E) with surrounding adipose tissue (A).

On ultrasound, an infiltrative hypoechoic lesion with two associated internal echogenic foci and extensive posterior acoustic shadowing was appreciated in the subcutaneous plane (Figure 1a). Minimal vascularity was appreciated on power Doppler (Figure 1b). The MR examination showed a similar sized spiculated mass, which appeared hypointense on T1W and T2W images with internal hyperintense foci on T1W fat supressed images suggestive of haemorrhage (Figures 2a, b and Figure 3a). The mass demonstrated restricted diffusion on DWI and heterogeneous enhancement on post-contrast images (Figures 2c, d and Figure 3a–b). It mainly involved the subcutaneous plane in left lower anterior abdominal wall and revealed poorly defined fat planes with the underlying rectus muscle, which, however, appeared normal in signal intensity.

The patient underwent wide local excision of the mass, which was removed with clear margins. The lesion was found to be adherent to the underlying rectus sheath (which was not infiltrated), measured 3 cm × 3 cm × 2.5 cm in size and showed scar tissue centrally with surrounding adipose tissue (Figure 3c). Microscopic analysis with haematoxylin and eosin (H&E) stain showed endometriotic glands and stroma with associated blood and adjacent adipose tissue, confirming scar endometriosis.

The patient was followed up on an out-patient basis and continues to remain asymptomatic.

### Case 2

A 32-year-old female complained of a 2-year history of debilitating lower abdominal pain in the region of her abdominal scar, which worsened during menstruation. She had a prior history of two lower segment Caesarean sections (LSCS), followed by a hysterotomy (at 6 months of gestation, for intrauterine foetal death). On examination, a healthy approximated transverse LSCS scar was seen and a tender nodule, approximately 2 cm × 2 cm in size, was palpated superiorly, 3 cm from the scar.

**FIGURE 4:**
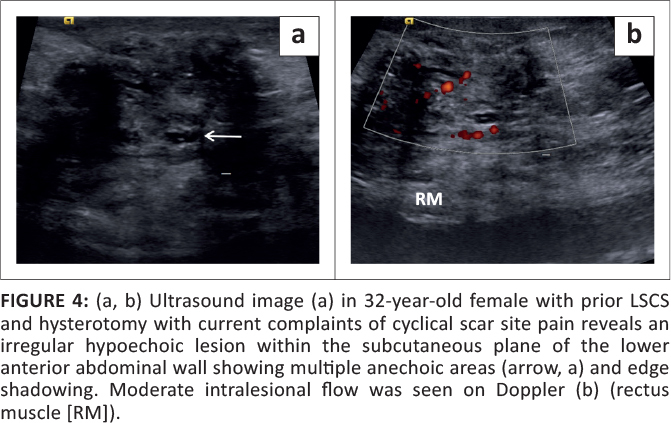
(a, b) Ultrasound image (a) in 32-year-old female with prior LSCS and hysterotomy with current complaints of cyclical scar site pain reveals an irregular hypoechoic lesion within the subcutaneous plane of the lower anterior abdominal wall showing multiple anechoic areas (arrow, a) and edge shadowing. Moderate intralesional flow was seen on Doppler (b) (rectus muscle [RM]).

**FIGURE 5:**
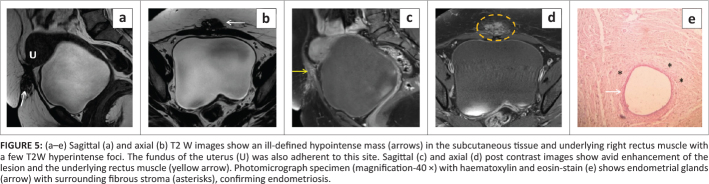
(a–e) Sagittal (a) and axial (b) T2 W images show an ill-defined hypointense mass (arrows) in the subcutaneous tissue and underlying right rectus muscle with a few T2W hyperintense foci. The fundus of the uterus (U) was also adherent to this site. Sagittal (c) and axial (d) post contrast images show avid enhancement of the lesion and the underlying rectus muscle (yellow arrow). Photomicrograph specimen (magnification-40 ×) with haematoxylin and eosin-stain (e) shows endometrial glands (arrow) with surrounding fibrous stroma (asterisks), confirming endometriosis.

Superficial anterior abdominal wall ultrasound revealed an ill-defined hypoechoic lesion with multiple internal anechoic areas and extensive edge shadowing (Figure 4a). Moderate vascular flow was observed on colour Doppler (Figure 4b). On MRI, an ill-defined mass was seen, the bulk of which appeared hypointense on T1W and T2W images and showed few T2W hyperintense foci within (Figure 5a, b). The lesion involved the subcutaneous and intramuscular planes (medial aspect of right rectus) (Figure 5c, d).

The patient consented for surgical excision of the lesion and a haemorrhagic nodule measuring 3 cm × 3.5 cm × 4 cm, which was partly infiltrating the right rectus muscle, was excised with clear margins. Histopathological analysis of the mass demonstrated the endometriotic nodule with adjacent adipose tissue, thus confirming scar endometriosis (Figure 5e).

The patient remained asymptomatic at follow up after three months.

### Case 3

A 24-year-old lady complained of continuous debilitating lower abdominal pain with no periodicity for one year prior to presentation. She had a past history of a LSCS two years back. Local examination revealed a healthy Pfannenstiel incision scar with surrounding tenderness; however, no obvious swelling was palpable.

**FIGURE 6:**
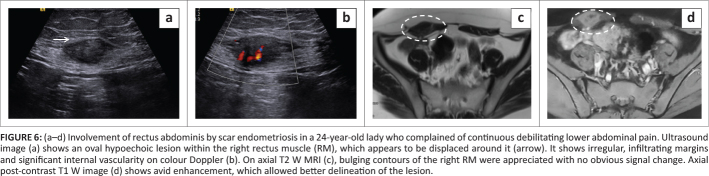
(a–d) Involvement of rectus abdominis by scar endometriosis in a 24-year-old lady who complained of continuous debilitating lower abdominal pain. Ultrasound image (a) shows an oval hypoechoic lesion within the right rectus muscle (RM), which appears to be displaced around it (arrow). It shows irregular, infiltrating margins and significant internal vascularity on colour Doppler (b). On axial T2 W MRI (c), bulging contours of the right RM were appreciated with no obvious signal change. Axial post-contrast T1 W image (d) shows avid enhancement, which allowed better delineation of the lesion.

Suspicion of scar endometriosis was first raised on sonography when evaluation (at the site of maximum tenderness) revealed an oval hypoechoic lesion, which showed irregular, infiltrating margins with internal vascularity on Doppler evaluation (Figure 6a, b). It involved the right rectus muscle with resultant contoural bulge and was situated about 5 cm above the scar. MRI confirmed the sonographic findings and showed an avidly enhancing oval mass. It was predominantly within the right rectus muscle, which showed bulging contours on conventional T1 and T2W images; however, no obvious signal change was appreciated (Figure 6c). This inconspicuous nature could be explained by the similar signal intensity of lesion to the rectus muscle. Avid enhancement on the post-contrast images helped in its delineation (Figure 6d).

At surgery, the mass was seen within the rectus muscle with extension into the subcutaneous plane. It was excised with clear margins and measured 3.5 cm × 2 cm × 2.5 cm in size. Microscopic examination showed features consistent with scar endometriosis. The patient continues to remain asymptomatic on routine outpatient follow up.

### Case 4

A 33-year-old female presented with cyclical pain and swelling in the right vulval region over two years. She had a normal vaginal delivery with a right-sided episiotomy seven years previously. On examination, a small tender nodule was felt on deep palpation in the perineal region at the 7 o’clock position.

**FIGURE 7:**
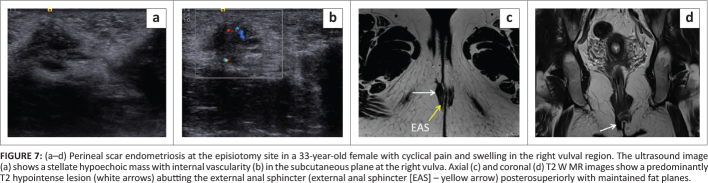
(a–d) Perineal scar endometriosis at the episiotomy site in a 33-year-old female with cyclical pain and swelling in the right vulval region. The ultrasound image (a) shows a stellate hypoechoic mass with internal vascularity (b) in the subcutaneous plane at the right vulva. Axial (c) and coronal (d) T2 W MR images show a predominantly T2 hypointense lesion (white arrows) abutting the external anal sphincter (external anal sphincter [EAS] – yellow arrow) posterosuperiorly with maintained fat planes.

**FIGURE 8:**
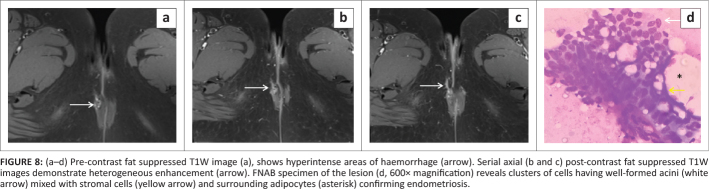
(a–d) Pre-contrast fat suppressed T1W image (a), shows hyperintense areas of haemorrhage (arrow). Serial axial (b and c) post-contrast fat suppressed T1W images demonstrate heterogeneous enhancement (arrow). FNAB specimen of the lesion (d, 600× magnification) reveals clusters of cells having well-formed acini (white arrow) mixed with stromal cells (yellow arrow) and surrounding adipocytes (asterisk) confirming endometriosis.

On B-mode ultrasonography, a stellate hypoechoic mass with internal vascularity was observed in the subcutaneous plane in the right vulval region (at the site of the palpable nodule) (Figure 7a, b). On MRI, the mass demonstrated spiculated margins and appeared hypointense on T1W and T2W images. A few hyperintense foci were observed on the T1W fat supressed images, suggestive of haemorrhages. The mass also showed diffusion restriction on DWI/ADC and heterogeneous enhancement on post-contrast images. The lesion involved the subcutaneous plane and was abutting the external anal sphincter on the right side with maintained intervening fat planes (Figure 7c, d and Figure 8a–c).

Ultrasound guided fine needle aspiration biopsy (FNAB) of the mass was performed, revealing epithelial cells, hemosiderin laden macrophages and spindle cells with fibroadipose tissue, which suggested scar endometriosis (Figure 8d). However, the patient refused surgery and was subsequently lost to follow up.

None of the patients in the series had concomitant endometriosis at any other site on imaging.

## Discussion

As defined by the presence of functional endometrial tissue (consisting of glands and stroma) outside the uterus,^5^ endometriosis is a polymorphic condition that can manifest as superficial implants on the peritoneal surface, ovarian cysts called endometriomas and deep lesions that infiltrate the peritoneal surfaces, known as deep infiltrative endometriosis (DIE). The common sites of involvement include the ovaries (most common), uterosacral ligaments, serosal surfaces, pouch of Douglas, fallopian tubes, rectosigmoid and urinary bladder. However, any tissue can potentially be involved by these endometriotic implants including the liver, small bowel, appendix, lung, brain, abdominal wall and perineum.

Abdominal or pelvic wall endometriosis consists of ectopic endometrial tissue embedded within the subcutaneous adipose layers and the muscles of the abdominal wall^6^ and is usually associated with scar tissue from a previous surgical procedure, most commonly a CS, with an estimated incidence of approximately 0.03% – 0.4%.^2,7,8^

Perineal scar endometriosis is a rare condition with an incidence of 0.03% – 0.15%^9^; however, it can cause severe morbidity because of close proximity to and hence higher potential for involvement of the anal sphincter.

The exact pathogenesis of scar endometriosis still remains to be elucidated; however, it is known that surgery remains a sine qua non inciting factor for this condition. Uterine manipulation during surgeries can potentially lead to dissemination and implantation of ectopic endometrium at surgical sites, which is known as the theory of metastatic implantation. It is further supported by the fact that many patients with scar endometriosis (including the cases in this series) do not have symptoms or signs of endometriosis because of dissemination of endometrial cells into the wound at the time of surgery. The metaplastic theory is an alternative theory which proposes the differentiation of coelomic stem cells into endometriotic tissue within the scar and hence may explain the presence of endometriosis at other sites. However, the metastatic theory is currently more favoured as a plausible cause for scar endometriosis.^3,10^

Even after complete excision, occasionally this condition can be recurrent, with a recurrence rate of nearly 15% for abdominal and 14% for perineal endometriosis, as shown in a recent study.^11^ Continuous recurrence following excision should raise the suspicion of malignant transformation, typically to endometrioid or clear cell carcinoma, which has a documented incidence of approximately 1%.^12^

The diagnosis of scar endometriosis is mostly made in women of reproductive age following instrumentation of the uterus.^13^ A presumptive clinical diagnosis may be made on the basis of a triad of clinical features, which include a history of surgery, cyclical pain at or near the scar site progressively increasing with menstrual cycles and a palpable, painful abdominal mass (as present in three patients of our series). However, as seen in case 3, a significant proportion of patients may present with continuous lower abdominal pain, dysmenorrhoea or may show no symptoms at all, with the findings being discovered incidentally. A variable latent period is also observed before the symptom onset which, in a recent study, was found to have a possible correlation with the depth of involvement.^6^

On histopathology, scar endometriosis consists of endometrial glands and stroma with intervening focal areas of chronic inflammation, fibrous tissue, smooth muscle hyperplasia and hemosiderin deposition. The presence of hemosiderin-filled macrophages is a peculiar feature seen in endometriosis.^3^

It should be realised that because the ectopic endometrial tissue remains responsive to hormonal stimulation, the appearance of scar endometriosis on imaging may vary depending upon the phase of the patient’s menstrual cycle, with maximum growth of the tissue in the late secretory and menstrual phase. This often also coincides with the severity of symptoms experienced by the patient. Hence, imaging should be scheduled either just prior to or during the menstrual phase with due consideration of the patient’s symptomology, which helps in correct assessment of the size and extent of the lesion.^14^

Irrespective of the site and modality, scar endometriosis is characteristically seen as an irregular nodule with spiculated infiltrative margins because of associated extensive fibrosis and chronic inflammation.

Sonography remains the first line for imaging of these lesions with emphasis on the use of high frequency transducers as they may be missed on examination by routine 3 MHz – 5 MHz transducers. While appearances are varied, ranging from solid, mixed solid-cystic or multicystic lesions, the most common appearance is of a solid inhomogeneous infiltrative hypoechoic mass with echogenic foci, spiculated margins and thick echogenic strands.^1,3,15^ As a result of the associated infiltration of the adjacent soft tissues, the lesions are often not clearly delineated from surrounding tissues, thus limiting the accuracy in assessing the lesion size and infiltration depth.^14^ Internal cystic changes may also be seen representing haemorrhagic foci or dilated ectopic glands. Contrary to endometriomas in the ovary, scar endometriosis often shows internal vascularity on colour Doppler with a single vascular pedicle or dilated feeding vessels at the periphery or centre of the mass.^2^

MRI is a non-invasive modality which allows good tissue characterisation and offers the advantage of multiplanar evaluation of the lesions.^3,16^ It provides an accurate assessment of the extent including depth of involvement (i.e. whether limited to the subcutaneous plane, intramuscular plane, peritoneal involvement etc.) by the endometriotic lesions and has high sensitivity for detection of blood products. As evident in cases 1 and 2 in our series, MRI demonstrated greater precision of depth assessment and showed better concordance with surgical findings. In addition, it can also identify endometriosis in other locations and hence MRI plays a crucial role in preoperative planning in order to achieve complete resection of the lesions, thereby minimising recurrence.^3^

As a result of its fibrotic nature and the presence of blood products of different ages, the bulk of the lesion appears heterogeneously hypointense on T1W and T2W images with some small T1 and T2 hyperintense foci within, representing areas of subacute haemorrhage, as seen in cases 1 and 4. Use of fat suppressed T1W images improves the conspicuity of such haemorrhagic foci leading to a significant increase in accuracy for lesion detection. Isolated T2 hyperintense foci, as observed in case 2 may also be seen, which represent ectopic glandular elements.^16,17^ Diffusion characteristics may vary depending on the predominant tissue within the lesion. On post-contrast images, the lesions invariably demonstrate intense enhancement (evident in all four cases) with an occasional feeding vessel sign, which has been attributed to the associated extensive inflammation.^3^ Hence contrast-enhanced-MRI forms an integral part for imaging of such lesions. In perineal scar endometriosis, preoperative MR imaging plays an indispensable role by identifying the involvement of the anal sphincter complex, which can significantly impact the surgical management.^9^

An excisional biopsy is the gold standard for diagnosis of this condition. However, in cases where FNAB or incisional biopsy are performed (as in case 4), excision of the needle tract is required at the time of surgical excision in order to prevent recurrence.

The ultimate curative treatment for these lesions is a wide excision with at least 1 cm clear margins to prevent recurrence and possible future malignant transformation.^3,14^ Deeper lesions often require skin flaps or wire mesh for covering the resultant fascial defects in abdominal wall endometriosis. Anal sphincter involvement in perineal scar endometriosis may require sphincter reconstruction. Careful manipulation of tissue is essential as mishandling during surgery can potentially lead to dissemination of implants and future recurrence. Post-surgery, patients may be treated with hormonal-based therapies such as gonadotropin releasing hormone (GnRH) and progesterone analogues, which cause suppression of the potentially retained microscopic implants thereby preventing recurrence. Although very rare, potential malignant transformation of the lesion needs consideration when dealing with continued recurrence.^14^

Radiologists should be aware of this entity especially in women of reproductive age with classical clinical and imaging findings. Other differential diagnoses that may be seen in the context of previous surgery and hence merit consideration are tabulated in Table 1.

**TABLE 1:** Differential diagnoses of surgical scar endometriosis.

Lesion	Location	Clinical features	Relevant imaging findings
Hematoma	Abdominal wall and perineum	Post surgery or trauma as an acute-subacute complication; no periodicity of symptoms.	Serial evolution of findings USG - initially hyperechoic then hypoechoic to anechoic MRI - varying T1 and T2 signal intensities due to evolution of blood products
Keloid/hypertrophic scars	Abdominal wall and perineum	Irregular lobulated bumpy growth (due to hyperplasia of fibrous tissue), typically extends beyond the confines of the original wound in keloid while remains within in hypertrophic scar. Accompanied with itching_;_ however usually non painful	USG - hypoechoic lesion with irregular infiltrative margins. MRI - T1 and T2 hypointense, delayed enhancement
Desmoid tumors	Abdominal wall	Association with Gardner’s syndrome. Non painful, progressively increasing palpable lump	USG - well demarcated, homogenously hypoechoic, solid masses with occasional infiltrative margins and lobulations CT - circumscribed masses with post contrast enhancement MRI - hypointense on T1 and T2, minimal to no significant enhancement
Suture granulomas	Abdominal wall and perineum	Palpable tender mass; no cyclical pain	Similar to scar endometriosis however key is to identify the retained suture material USG - ill-defined hypoechoic lesion with hyperechoic parallel strands within CT - linear hyperdense suture material with occasional calcifications within. MRI - variable
Incisional hernia	Abdominal wall	Reducible swelling; increasing on coughing and straining 2. Painless unless irreducible	USG/ CT/ MRI - presence of a wall defect with herniation of the abdominal viscera through it

## Conclusion

In a female of reproductive age with previous pelvic surgery complaining of cyclical abdominal pain with swelling at or near the surgical site, a diagnosis of scar endometriosis is of prime concern. Radiology plays an important role in its accurate detection and also excludes the various differential diagnoses. Besides confirming the diagnosis, imaging, especially MRI, evaluates the extent of tissue involvement by the endometriotic lesion and helps the surgeon in devising an appropriate patient centred management strategy.
